# Transgenerational effects of temperature fluctuations in *Arabidopsis thaliana*

**DOI:** 10.1093/aobpla/plab064

**Published:** 2021-10-11

**Authors:** Ying Deng, Oliver Bossdorf, J F Scheepens

**Affiliations:** 1 Institute of Evolution and Ecology, University of Tübingen, Tübingen 72076, Germany; 2 Natural History Research Center, Shanghai Natural History Museum, Shanghai 200041, China; 3 Faculty of Biological Sciences, Goethe University Frankfurt, Frankfurt am Main 60438, Germany

**Keywords:** Environmental variability, genetic variation, heat stress, natural variation, phenotypic plasticity

## Abstract

Plant stress responses can extend into the following generations, a phenomenon called transgenerational effects. Heat stress, in particular, is known to affect plant offspring, but we do not know to what extent these effects depend on the temporal patterns of the stress, and whether transgenerational responses are adaptive and genetically variable within species. To address these questions, we carried out a two-generation experiment with nine *Arabidopsis thaliana* genotypes. We subjected the plants to heat stress regimes that varied in timing and frequency, but not in mean temperature, and we then grew the offspring of these plants under controlled conditions as well as under renewed heat stress. The stress treatments significantly carried over to the offspring generation, with timing having stronger effects on plant phenotypes than stress frequency. However, there was no evidence that transgenerational effects were adaptive. The magnitudes of transgenerational effects differed substantially among genotypes, and for some traits the strength of plant responses was significantly associated with the climatic variability at the sites of origin. In summary, timing of heat stress not only directly affects plants, but it can also cause transgenerational effects on offspring phenotypes. Genetic variation in transgenerational effects, as well as correlations between transgenerational effects and climatic variability, indicates that transgenerational effects can evolve, and have probably already done so in the past.

## Teaser

When plants experience stressful environmental conditions, such as heat waves, this can even affect their offspring, a phenomenon called ‘transgenerational effects’. We were interested in how important the precise temporal patterns of heat stress are, and we therefore subjected *Arabidopsis thaliana* plants to a range of different heat stress regimes. We find that the timing of heat events is much more important than their frequency. We also find that the transgenerational responses of plants to heat are related to their climates of origin, which means they can evolve, and have probably already done so in the past.

## Introduction

Plants encounter various environmental challenges in nature, such as episodes of stressful temperatures or low water availability. Many previous studies have investigated how plants respond to contrasting environmental conditions in terms of their fitness and functional traits (e.g. Sultan *et al.* 1998; Callahan and Pigliucci 2002; Ibañez *et al.* 2017; Marais *et al.* 2017). Although plants generally show reduced fitness under stressful environments, different genotypes often vary in their fitness responses and thus their ability to maintain fitness under adverse environmental conditions (Sultan 1987, 2000; Ghalambor *et al.* 2007). This variation in fitness responses is often related to underlying variation in the plasticity of functional traits. For instance, decreased fitness under warmer temperatures may be caused by advanced flowering in the annual *Arabidopsis thaliana* (Ibañez *et al.* 2017). More generally, there is usually intraspecific variation in plant responses to environmental treatments (i.e. genotype by environment interactions, G × E; Sultan 2000; Pigliucci 2001). If such variation exists within populations, then natural selection can act on it, and the trait plasticity can evolve and adapt to local environmental conditions (Sultan 2000; Groot *et al.* 2017). If past environments have influenced the evolution of plasticity, we should be able to detect plasticity–environment correlations to identify agents of selection shaping plasticity (Groot *et al.* 2017; Marais *et al.* 2017).

Organisms may not only respond directly to their current environments, but their phenotypes may also be influenced by the environmental conditions that their ancestors were exposed to (Uller 2008; Latzel *et al.* 2014; Groot *et al.* 2016, 2017; Alvarez *et al.* 2020; Liu *et al.* 2020)—a phenomenon called ‘transgenerational plasticity’ or ‘transgenerational effects’. In plants, such transgenerational effects can be physiological and controlled by the mother plant (Herman and Sultan 2011), for instance through endosperm or seed coat modifications. Transgenerational effects can also be epigenetic (Whittle *et al.* 2009; Rasmann *et al.* 2012; Suter and Widmer 2013) and therefore potentially transferable across even more than one generation (Suter and Widmer 2013; Groot *et al.* 2016, 2017). Through transgenerational effects, plants could prepare (or ‘prime’) their phenotypes for particular environmental conditions, particularly when offspring are likely to experience similar conditions as their parents, thereby increasing local adaptation (i.e. adaptive transgenerational plasticity; Roach and Wulff 1987; Mousseau and Fox 1998a, b; Agrawal 2001; Galloway 2005; Galloway and Etterson 2007; Uller 2008; Mousseau *et al.* 2009; Latzel *et al.* 2014; Yin *et al.* 2019; Puy *et al.* 2021). However, as with regular (within-generation) phenotypic plasticity, transgenerational effects can only evolve as an adaptation when there is genotypic variation in transgenerational effects and when offspring environmental conditions correlate with parental environmental conditions (Uller 2008).

An increasing number of empirical studies with plants investigated how transgenerational effects may confer adaptation particularly under temperature stress (Sultan *et al.* 2009; Herman and Sultan 2011; Latzel *et al.* 2014; Groot *et al.* 2017). For instance, in a single genotype of the model plant *A. thaliana*, transgenerational effects of heat stress were observed even in the third offspring generations (Whittle *et al.* 2009). Interestingly, the third offspring generations which experienced the same heat stress in the parental generation and first offspring generations had a fitness advantage. More recently, Groot and co-workers (2017) showed strong genotypic variation in parental and grandparental effects of heat stress in 14 *A. thaliana* genotypes.

So far most studies investigating plant responses to altered and/or stressful environmental conditions—including those studies investigating transgenerational effects—were performed under controlled conditions. However, studies usually applied stable treatments that did not consider the temporal variability of environmental stress, which however plays an important role in natural ecosystems (Knapp *et al.* 2002; Schwinning *et al.* 2004; Shea *et al.* 2004). For instance, while global warming is expected to continue (Giorgi *et al.* 2004; Barros and Field 2014), climate anomalies will increase too (e.g. European heat waves in 2003 and 2010), resulting in increasing temporal variability of temperature and, presumably, heat stress (Schär *et al.* 2004; Fischer and Schär 2008; Barriopedro *et al.* 2011). During climatic extreme events, the variability aspect itself is often thought to be more important than the involved changes in means (Katz and Brown 1992), and some ecosystems have even been found to be more sensitive to changes in environmental variability than to changes in environmental means (Knapp *et al.* 2002).

To date, only few studies have examined plant responses to changes in environmental variability, or genetic variation therein (Parepa *et al.* 2013; Scheepens *et al.* 2018), specifically with respect to the timing (Stone and Nicolas 1995, 1996; Prasad *et al.* 1999; Wang *et al.* 2016) or frequency (Walter *et al.* 2009) of stress. To our knowledge, only one previous study tested for transgenerational effects of stress timing (Reza Rahavi and Kovalchuk 2013) and none tested transgenerational effects of stress frequency.

To address these questions and to better understand the complexity of plant responses to climatic variability (Knapp *et al.* 2002; Reyer *et al.* 2013), we carried out a two-generation experimental study with *A. thaliana* that tested plant responses to altered timing and frequency of heat stress. To explore intraspecific variation and evolutionary potential, our study included multiple genotypes from different geographic and climatic origins. In the first generation (published in Scheepens *et al.* 2018), we found (i) that the timing of heat stress had a much stronger effect on the plants than its frequency, (ii) that *A. thaliana* genotypes significantly differed in their responses to stress timing and (iii) that this intraspecific variation correlated with the precipitation variability at the geographic origins. The latter two findings together indicate a possible adaptive evolution of this type of phenotypic plasticity in more variable environments.

Here, we report on the results from the offspring generation where we grew plants from 9 of the 11 genotypes included in the parental-generation experiment in two experiments: On the one hand we tested for transgenerational effects of parental stress treatments in a simple common-garden experiment, and on the other hand we subjected a subset of the offspring plants to renewed stress to test the adaptive value of transgenerational effects (reciprocal experiment). As in the parental-generation experiment, we also tested for intraspecific variation in plant responses, correlated this variation with climates of origin and tested whether increased trait plasticity correlates with fitness robustness, i.e. more stable fitness across treatments. Specifically, we asked the following questions: (i) Are there transgenerational effects of heat stress timing or frequency on the phenotypes of the offspring? (ii) If yes, do transgenerational effects affect responses to current stress in an adaptive way? (iii) Are there differences among *A. thaliana* genotypes in the magnitudes and/or direction of transgenerational effects? (iv) If yes, does this intraspecific variation correlate with environmental conditions at the geographic origins and/or with fitness robustness?

## Materials and Methods

### Parental-generation experiment

**Figure 1.**
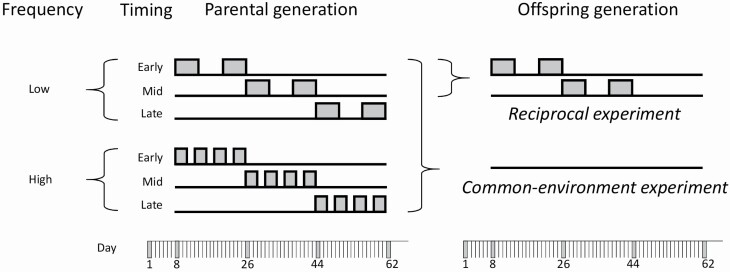
Experimental design of the parental-generation experiment (left) and the two offspring experiments (right) with *Arabidopsis thaliana*, with periods of 30 °C heat stress indicated in grey. In the offspring generation, plants from all parental treatments are grown in a constant control environment (common-environment experiment), and plants from two parental stress treatments are subjected to the same two treatments again (reciprocal experiment).

The plant material used here came from a previous study (Scheepens *et al.* 2018) in which we tested for the direct effects of different temperature stress scenarios, varying in timing and frequency (Fig. 1), on 11 *A. thaliana* genotypes. The 11 genotypes were selected to maximize genetic diversity and came from the ‘core collection’ of the Versailles Arabidopsis Stock Center (McKhann *et al.* 2004). After 1 week of cold-moist (4 °C) stratification, all seeds were planted into 5 × 5 × 4.5 cm pots with a 9:9:2 mixture of low-nutrient soil, regular potting soil and sterilized sand and placed in a growth chamber with 20/15 °C and a 16/8 h light/dark cycle until 1 week after germination. For the experimental treatments, we used two identical climate chambers, one set to 20/15 °C (‘control chamber’), the other set to 30/25 °C (‘stress chamber’), both with a 16/8 h light/dark cycle. A day temperature of 30 °C is known to be stressful for *A. thaliana* and to reduce its fitness (Groot *et al.* 2017; Scheepens *et al.* 2018). Light conditions (230 μmol⋅m^−2^⋅s^−1^) and air humidity (40–60 %) were identical in both chambers. The experimental treatments were created by moving different subsets of plants to the stress chamber at different times and intervals. Specifically, we varied the timing and frequency of heat stress periods experienced by the plants (Fig. 1). To vary timing, we stressed plants either early in their life cycle (plants moved to stress chamber on Day 8, right after the first week of seedling establishment), in the middle of most genotypes’ life cycle (starting on Day 26) or late in the life cycle (starting on Day 44). The timing treatment was crossed with a frequency/duration treatment, where heat stress was either applied at low frequency (2 times 6 days of stress, with 6 days in between) or high frequency (4 times 3 days of stress, each time with 2 days in between). Important to note is that in all stress scenarios the plants experienced the same total time in the stress chamber and therefore also the same mean temperature during the experiment (Fig. 1). In each chamber, the spatial positions of all pots were completely randomized, and were re-randomized every week. We had eight replicate plants of each genotype in each treatment. Altogether, our parental-generation experiment included 11 genotypes × 6 treatments × 8 replicates = 528 plant individuals. The experiment ran for ~10 weeks. When plants began flowering, we placed their inflorescences into ARACON tubes (Betatech bvba, Gent, Belgium) to prevent cross-fertilization and collect the seeds for the next experimental generation.

### Offspring generation experiments

We tested for transgenerational effects in two separate experiments, (i) a simple common-environment comparison of offspring from the six parental treatments under control condition (16/8 h light/dark at 20/15 °C), and (ii) a reciprocal transplant where we used offspring from only two of the parental treatments, the early and mid-term stress at low stress frequency (Fig. 1), re-created these two treatments and grew both types of offspring in both environments. We restricted the second experiment to these two treatments because they had the strongest effects in the parental generation (Scheepens *et al.* 2018). Since in the reciprocal experiment there were ‘local’ (same conditions as parents) versus ‘foreign’ (different conditions from parents) plants in each environment, this experiment allowed to test for adaptive transgenerational effects. In both offspring experiments, we used 9 of the 11 genotypes from the previous generation, because of limited numbers of seeds in the remaining two genotypes (Table 1; Scheepens *et al.* 2018), and we stratified and germinated seeds as in the parental experiment. In the first experiment, we had seven replicates per genotype and parental treatment, for a total of 9 genotypes × 6 parental environments × 7 replicates = 378 plants. In the second experiment, there were eight replicates per genotype by treatment combination, with a total of 9 genotypes × 2 parental environments × 2 offspring environments × 8 replicates = 288 plants. In both experiments, we watered all plants regularly, and re-randomized their spatial positions every week. On Day 44, right after the intermediate stress treatment in the reciprocal experiment, we took leaf samples for molecular analyses (not reported here) from 3 to 4 randomly selected plants from each genotype by treatment combination in each of the two experiments (i.e. from roughly half of the plants). Throughout the experiment, we recorded flowering time as the number of days from germination to when the white petals of the first flower became visible. As in the parental experiment, we placed ARACON tubes over the flowering stems to prevent outcrossing and collect seeds. Each plant was harvested 1 week after its fruits had started to turn yellow. We estimated plant fecundity as the number of fruits >2 mm. We then counted the number of basal shoots and lateral shoots and calculated the ratio of lateral to basal shoot number as index of plant architecture, with lower values indicating more ‘shrubby’ plants. After that, we separated inflorescences and rosettes, dried them at 60 °C for 72 h and weighed them, and then calculated total above-ground biomass, as well as reproductive allocation as the ratio of reproductive to total above-ground biomass.

**Table 1.** *Arabidopsis thaliana* genotypes used in this study, and their geographical coordinates and natural growing season (in months; from Scheepens *et al.* 2018).

Name	Country	Latitude	Longitude	Growing season
Bur-0	Ireland	54.1	−6.2	5–8
Can-0	Spain	29.21	−13.48	11–2
Ct-1	Italy	37.51	15.09	12–3
JEA	France	43.68	7.33	3–6
Mt-0	Libya	32.34	22.46	11–2
N13	Russia	61.36	34.15	6–9
Oy-0	Norway	60.39	6.19	5–8
Sha	Tajikistan	38.59	68.79	2–5
St-0	Sweden	59.34	18.06	5–8

### Statistical analysis

We used linear models to test for the effects of experimental treatments, plant genotypes, and their interactions, on each of the five measured traits: flowering time, plant architecture, above-ground biomass, reproductive allocation and fecundity, where fecundity is interpreted as a fitness proxy. For the simple common-environment experiment, the models included plant genotype, timing of parental stress, frequency of parental stress and all possible interactions, as fixed factors. For the reciprocal experiment, the models included plant genotype, timing of parental stress, timing of offspring stress and their interactions. Additionally, to account for possible influences of the leaf sampling, all models also included leaf sampling (yes/no) as a fixed factor. To improve the normality of residuals and homogeneity of variance, the flowering time and above-ground biomass data were log-transformed prior to the analyses. Despite these transformations, Levene’s tests showed that the assumption of homogeneity of variance was violated for significant interactions that included genotype, possibly due to low sample sizes at this interaction level. However, linear models are fairly robust to heteroscedasticity when sample sizes are equal, which is the case in our study.

In those cases where we discovered a significant genotype by treatment interaction, i.e. genetic variation in plasticity, in either of the two experiments, we additionally tested whether trait plasticities of genotypes were associated with (i) their climates of origin and (ii) their fitness robustness. As measure of trait plasticity, we used the coefficient of variation (CV) of a trait (Valladares *et al.* 2006) across all treatments in an experiment (common environment: six parental environments; reciprocal experiment: four combinations of parental and offspring environments). For the climate–plasticity test, we extracted climate data for each genotype origin from the WorldClim database (Hijmans *et al.* 2005), and we used on the one hand several existing bioclimatic variables that describe annual climatic variability [BIO_2_ = Annual Mean Diurnal Temperature Range, BIO_3_ = Isothermality, BIO_4_ = Temperature Seasonality (standard deviation, SD), BIO_7_ = Annual Temperature Range, BIO_15_ = Precipitation Seasonality (CV)], and on the other hand we calculated several climate variabilities for the specific growing season (see Table 1) of each genotype: the SDs of temperature, and the CVs of precipitation, evapotranspiration and climatological water deficit. Additionally, we included latitude from each genotype’s origin. To test for relationships between climate variability of origin and the plasticity of *Arabidopsis* genotypes, we calculated Pearson correlations between trait plasticity and the bioclimatic variables, growing-season variabilities and latitude, respectively. For the plasticity–fitness test, we calculated the fitness robustness of each genotype by taking the mean of the average fitness values (in terms of number of fruits) per treatment and by dividing this mean by the maximum average fitness achieved in one of the treatments. Fitness robustness thus indicates how treatments diminished fitness compared to maximum average fitness achieved among the treatments and allows for comparisons among genotypes (Scheepens *et al.* 2018). We then calculated Pearson correlations between trait plasticity and fitness robustness.

All statistical analyses were done in JMP 12 (SAS Institute, Heidelberg).

## Results

### Common-environment experiment

**Figure 2.**
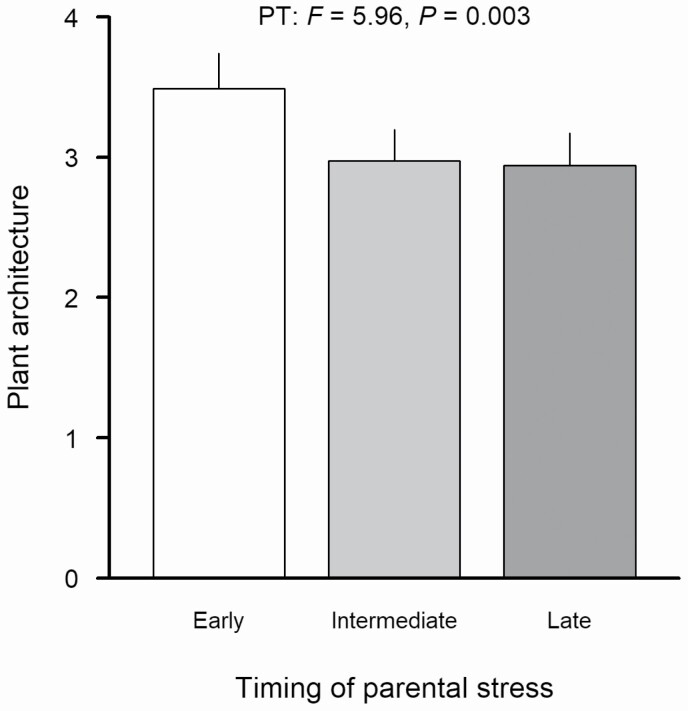
Effects of parental stress timing on plant architecture (number of lateral shoots/number of basal shoots) of *Arabidopsis thaliana* in the common-environment experiment. Error bars indicate SE. PT—Parental stress timing.

**Figure 3.**
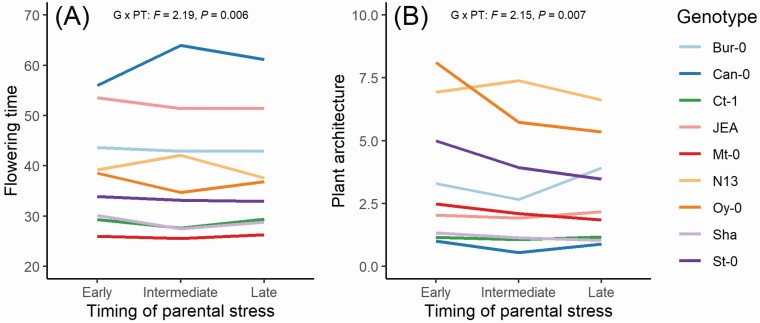
Genotypic variation in the transgenerational responses of flowering time (days since germination; A) and plant architecture (number of lateral shoots/number of basal shoots; B) of nine *Arabidopsis thaliana* genotypes to different timing of parental heat stress in the common-environment experiment. G × PT—genotype by parental stress timing interaction.

In the simple common-environment experiment, we found strong genotype differences in all measured traits (Table 2), confirming that there was substantial genetic diversity in the studied *A. thaliana* genotypes. The effects of parental stress treatments were much more moderate, and were largely confined to the timing of parental heat stress (Table 2; **see****Supporting Information—Table S4**): Offspring from parents which experienced early stress generally showed an increased ratio of lateral to basal shoots compared to intermediate and late stress (Fig. 2). For flowering time, the effect of stress timing depended on stress frequency (PT × PF interaction in Table 2; **see****Supporting Information—Fig. S1**). We found significant genotype by stress timing interactions for flowering time and plant architecture (G × PT interactions in Table 2; Fig. 3), indicating genetic variation in these transgenerational responses. There were no main effects of stress frequency in any of the studied traits, and no genotype by stress frequency interactions. Only for above-ground biomass, there was a significant three-way interaction between plant genotype, parental stress timing and parental stress frequency (G × PT × PF interaction in Table 2), indicating complex relationships between these three factors. The removal of leaves from around half of the plants, which we accounted for by including it in our models, had strong effects on three out of five analysed traits (above-ground biomass, reproductive allocation, fecundity; Table 2).

**Table 2.** Results of the common-environment experiment, testing the effects of leaf sampling, parental stress timing, parental stress frequency, genotype and their interactions, on the flowering time, plant architecture, above-ground biomass, reproductive allocation and fecundity of *Arabidopsis thaliana* offspring. Significant effects (*P* < 0.05) are in bold; df = degrees of freedom.

		Flowering time		Plant architecture		Above-ground biomass		Reproductive allocation		Fecundity	
	df	*F*-ratio	*P*-value	*F*-ratio	*P*-value	*F*-ratio	*P*-value	*F*-ratio	*P*-value	*F*-ratio	*P*-value
Leaf sampling	1	1.03	0.311	1.41	0.236	52.88	**<0.001**	20.54	**<0.001**	32.43	**<0.001**
Parental timing (PT)	2	0.85	0.429	5.96	**0.003**	0.25	0.777	1.35	0.261	1.33	0.267
Parental frequency (PF)	1	0.95	0.331	2.82	0.094	0.33	0.567	0.25	0.615	1.06	0.305
PT × PF	2	5.92	**0.003**	0.12	0.891	0.19	0.831	0.55	0.577	0.16	0.852
Genotype (G)	8	260.23	**<0.001**	99.12	**<0.001**	35.65	**<0.001**	174.37	**<0.001**	79.23	**<0.001**
G × PT	16	2.19	**0.006**	2.15	**0.007**	1.30	0.193	1.29	0.202	1.19	0.275
G × PF	8	0.40	0.920	0.54	0.829	1.22	0.287	0.88	0.536	1.30	0.242
G × PT × PF	16	0.97	0.494	1.01	0.441	1.99	**0.013**	1.47	0.109	1.10	0.353

### Reciprocal experiment

**Figure 4.**
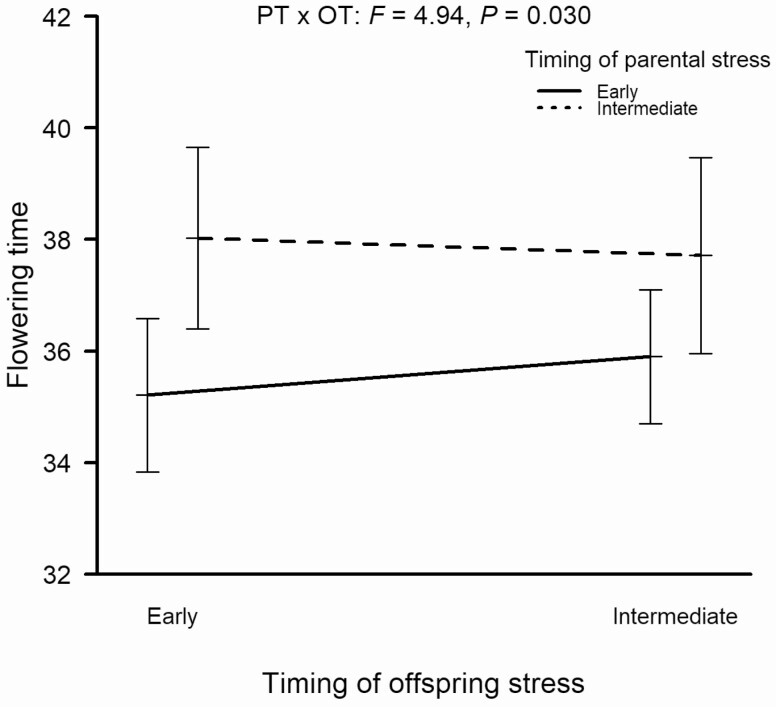
Effects of parental and offspring heat stress timing on flowering time (days since germination) in *Arabidopsis thaliana* in the reciprocal experiment. Error bars indicate SE. PT × OT—parental stress timing by offspring stress timing interaction.

When offspring from early and intermediate (low-frequency) stress parents were reciprocally subjected to the same treatments, there were strong effects of offspring environment on all measured traits except for flowering time (OT main effects in Table 3; **see****Supporting Information—Table S5**). The parental heat stress timing affected only the flowering time of the plants (PT main effect in Table 3; **see****Supporting Information—Table S5**), with offspring from early stress parents flowering earlier (Fig. 4). However, a significant interaction between parental and offspring environment (PT × OT in Table 3; **see****Supporting Information—Table S5**) indicated that the expression of transgenerational effects on flowering time depended on the offspring environment: the differences between parental treatments were expressed only if the offspring was subjected to early heat stress, but not if heat stress occurred later (Fig. 4).

**Table 3.** Results of the reciprocal experiment, testing the effects of leaf sampling, parental stress timing, offspring stress timing, genotype and their interactions, on the flowering time, plant architecture, above-ground biomass, reproductive allocation and fecundity of *Arabidopsis thaliana* offspring. Significant effects (*P* < 0.05) are in bold; df = degrees of freedom.

		Flowering time		Plant architecture		Above-ground biomass		Reproductive allocation		Fecundity	
	df	*F*-ratio	*P*-value	*F*-ratio	*P*-value	*F*-ratio	*P*-value	*F*-ratio	*P*-value	*F*-ratio	*P*-value
Leaf sampling	1	0.00	0.960	0.14	0.707	18.38	**<0.001**	7.90	**0.005**	11.88	**0.001**
Parental timing (PT)	1	9.92	**0.002**	0.00	0.970	0.21	0.651	0.14	0.708	2.07	0.152
Offspring timing (OT)	1	0.76	0.385	8.08	**0.005**	41.77	**<0.001**	114.43	**<0.001**	17.48	**<0.001**
PT × OT	1	4.74	**0.030**	0.01	0.914	0.23	0.630	0.84	0.360	0.21	0.643
Genotype (G)	8	184.29	**<0.001**	14.67	**<0.001**	12.13	**<0.001**	158.91	**<0.001**	57.10	**<0.001**
G × PT	8	3.50	**0.001**	0.50	0.856	0.90	0.517	1.17	0.317	0.86	0.549
G × OT	8	2.07	**0.039**	2.91	**0.004**	5.49	**<0.001**	1.59	0.128	3.97	**<0.001**
G × PT × OT	8	1.82	0.074	0.37	0.937	0.43	0.905	1.28	0.253	2.39	**0.017**

As in the common-environment experiment, leaf removal had significant effects on three out of five traits (above-ground biomass, reproductive allocation, fecundity), and there were significant genotype differences in all of the studied traits (Table 3). There were also significant genotype by offspring environment interactions (G × OT in Table 3) in four out of the five measured traits, indicating genetic variation in (within-generation) phenotypic plasticity. In addition, we found a genotype by parental environment interaction (G × PT in Table 3), indicating genotype-specific transgenerational effects, for flowering time.

We did not find a significant parental by offspring environment interaction for plant fecundity (PT × OT in Table 3; **see****Supporting Information—Table S5**), as would have been predicted for adaptive transgenerational effects. However, there was a significant G × PT × OT interaction, indicating that these interactions are genotype-specific. We therefore tested for a significant PT × OT interaction separately for each genotype. Only in Mt-0 this interaction was significant (*F* = 10.38, *P* = 0.003; **see****Supporting Information—Fig. S2**), but the results did not confirm our hypothesis. In each offspring environment the plants from the respective *other* parental environment produced more fruits than the ones from the same parental environment, indicating a maladaptive transgenerational effect.

### Plasticity, climates of origin and fitness robustness

**Figure 5.**
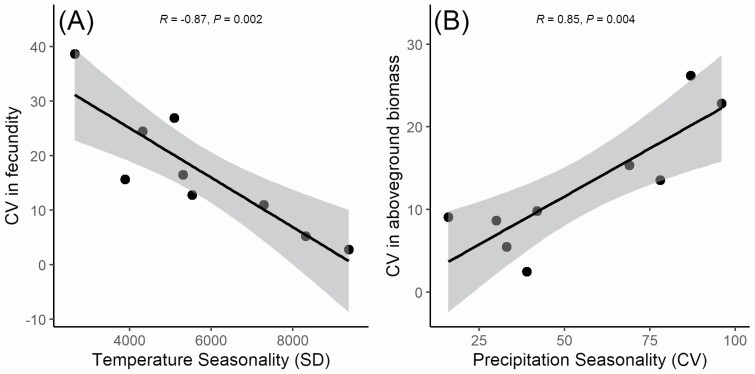
Relationships between trait plasticities and climates of origins for nine *Arabidopsis thaliana* genotypes in the reciprocal experiment. (A) Correlation between temperature seasonality (SD) and CV of fecundity. (B) Correlation between precipitation seasonality (CV) and CV in above-ground biomass. The CVs are calculated across experimental treatments. The grey areas indicate the 90 % confidence intervals of the correlations.

**Figure 6.**
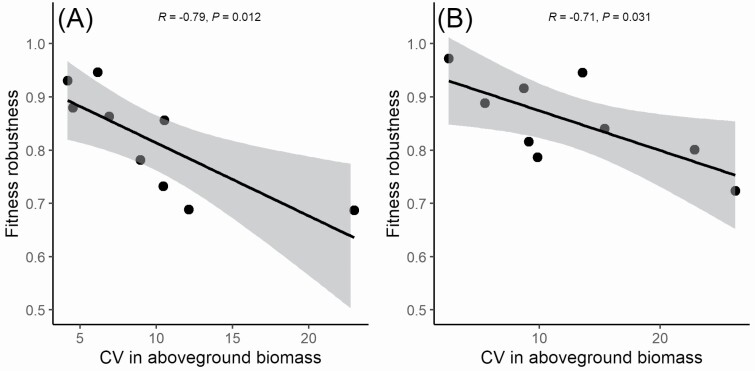
Relationships between fitness robustness across environments (see main text) and CV in above-ground biomass for nine genotypes of *Arabidopsis thaliana* in the common-environment experiment (A) and in the reciprocal experiment (B). The grey areas indicate the 90 % confidence intervals of the correlations.

We found no correlations between climates of origin and trait plasticity in the common-environment experiment **[see****Supporting Information—Table S1****]**, but in the reciprocal experiment there were several significant climate–plasticity correlations **[see****Supporting Information—Table S2****]**. The CV of fecundity (representing variation in fitness) was negatively correlated with temperature seasonality and annual temperature range, and positively correlated with isothermality **[see****Supporting Information—Table S2****]**. Thus, genotypes from geographic origins with higher temperature seasonality displayed lower variation in fecundity—and therefore greater fitness homeostasis—in response to different stress treatments (Fig. 5A). The CV of fecundity was also positively correlated with the seasonal CV of evapotranspiration variability **[see****Supporting Information—Table S2****]**. Moreover, we also found that the CV of above-ground biomass was positively correlated with isothermality and precipitation seasonality (Fig. 5B), and negatively correlated with latitude and with the seasonal CV of climatological water deficit. Finally, the CV of plant architecture correlated negatively with the annual mean diurnal temperature range. Despite significant genotypic variation in the response of flowering time to parental or offspring stress timing, this variation in plasticity did not correlate with any of the climate variables. In both experiments, we found that the plasticity of above-ground biomass, but not that of the other traits, was significantly negatively correlated with fitness robustness (**see****Supporting Information—Table S3**; Fig. 6).

## Discussion

Changes in the temporal variability of environmental stresses are an important aspect of climate change, but we so far know little about the evolutionary consequences for plants: (i) whether plant responses can be transgenerational, (ii) if plants harbour intraspecific variation (and thus evolutionary potential) in this respect and (iii) how such transgenerational responses relate to environmental adaptation and fitness. While previous studies usually compared stressed and non-stressed plants (e.g. Galloway and Etterson 2007; Herman *et al.* 2012; Groot *et al.* 2017), we manipulated the temporal patterns of heat stress, i.e. when the stress occurred and how it was apportioned across time, whereas the total amount of stress (i.e. temperature sums) was identical in all parental environments. Our study demonstrates that changes in the temporal patterns of heat stress can carry over to the next generation in *A. thaliana*, and that there is substantial genotypic variation in the magnitude and direction of these transgenerational effects. Reza Rahavi and Kovalchuk (2013) also manipulated heat stress timing in *A. thaliana* and found transgenerational effects: offspring from parents that were stressed at Day 7 after germination increased stem length and fresh weight compared to control plants after renewed heat stress, while offspring from parents that were stressed at Day 21 decreased stem length. Whereas they investigated a single genotype, our study reveals significant genotypic variation in transgenerational effects for some traits. Thus, changes in heat stress patterns not only affect plants directly (Scheepens *et al.* 2018), but also across generations, and these effects vary among genotypes. Still, in terms of variance explained the genotype main effects in our study were ~1 order of magnitude larger than the main effects and interactions of our experimental treatments, highlighting the substantial phenotypic variation among the studied *Arabidopsis* genotypes.

### Transgenerational effects of stress timing versus frequency

Overall, the timing of heat stress had much stronger transgenerational effects than its frequency, consistent with our observations in the parental plants (Scheepens *et al.* 2018). Variation in parental stress timing consistently affected the architecture, and, depending on the genotype and/or stress frequency, also the flowering time and biomass of offspring plants, whereas the transgenerational effects of stress frequency were only minor.

One possibility why stress frequency may play such a little role within and across generations is that plant physiological responses to heat stress may be triggered by the initial stress event, and simply remain ‘switched on’ afterwards, so that the number or duration of stress events does not matter, at least on the short timescales of our experiment. A candidate mechanism for this would be heat shock proteins that plants produce to stabilize protein function (Vierling 1991; Sung *et al.* 2003), and that may protect plants and their offspring against subsequent heat stress events. However, heat shock proteins are generally only activated when temperatures exceed 32 °C (Vierling 1991), whereas the maximum temperature in our experiments was 30 °C. An alternative explanation is that many physiological pathways that induce plastic responses may rely on integration over time, which, in our design with a constant total amount of stress, would lead to similar responses between frequency treatments.

In contrast to stress frequency, the timing of parental heat stress influenced several traits of the plant offspring. It is generally well-established that the susceptibility of many plant traits to environmentally induced developmental changes depends on the life stage. For instance, heat stress during floral bud development determines peg number in peanut (Prasad *et al.* 1999); in wheat the maximum sensitivity to heat stress for protein accumulation is during the grain filling period (Stone and Nicolas 1996); and in the herbaceous plants *Andropogon gerardii* and *Solidago canadensis* late-season heat stress causes the greatest reduction in photosynthetic productivity (Wang *et al.* 2016). The usual explanation for such results is that signalling pathways determining trait changes may be more sensitive during certain developmental phases (e.g. early in plant or organ development) than during other phases (e.g. late in plant or organ development). In our experiment, early heat stress occurred at a small seedling stage of *A. thaliana*, whereas in the intermediate treatment the plants were already much larger and well-established. In fact, some were already bolting and/or close to flowering. It is not surprising that heat stress effects differed between these plants. However, all arguments so far, as well as the empirical studies mentioned above, are about within-generation responses to heat stress, whereas in our study we examined transgenerational effects of the timing of heat stress (see also Reza Rahavi and Kovalchuk 2013). Thus, signalling and developmental regulation alone cannot explain our results, and there must be additional, so far unknown, physiological (Herman and Sultan 2011) and/or epigenetic (Whittle *et al.* 2009; Rasmann *et al.* 2012), mechanisms involved.

### No evidence for adaptive transgenerational plasticity

In the reciprocal experiment, we applied stress treatments to offspring plants to test if transgenerational effects can be adaptive. An adaptive transgenerational effect would generally be indicated by higher fitness in offspring that experienced the same environment as their parents compared to offspring that did not. We found that responses in plant fecundity (i.e. variation in fitness) to current stress timing depended on parental stress timing, but in a highly genotype-specific manner. In fact, the majority of the parent–offspring interactions for separate genotypes were non-significant and only the genotype Mt-0 showed a significant interaction to parental and offspring heat stress timing. However, the pattern was maladaptive, i.e. in contrast to our expectation offspring from parents with the same stress timing had a *lower* fitness. This contrasts with observations of adaptive transgenerational plasticity from previous studies (Galloway and Etterson 2007; Latzel *et al.* 2014). The virtual absence of significant interactions across genotypes in our study may have various explanations, such as limited within-population genetic variation in parental effects for the environment to select on, or a lack of selective pressure for adaptive responses under unpredictable temperature stress events.

Offspring plants that received early stress showed accelerated flowering when their parents had also experienced early stress compared to plants whose parents had experienced intermediate stress. Such advanced flowering may reflect an escape strategy (Franks 2011), which could enhance the possibility of lineage survival under continuing high temperature conditions (Wahid *et al.* 2007). Although the induction of earlier flowering by environmental stress treatments is known from previous studies (Balasubramanian *et al.* 2006; Franks 2011; Ibañez *et al.* 2017), its transgenerational aspect has so far been rarely studied. Suter and Widmer (2013) found phenotypic trait changes indicating accelerated flowering of *A. thaliana* in the fourth generation after three generations of heat exposure, but this effect disappeared after the second generation without heat exposure. Groot and co-workers (2017) observed earlier flowering in response to grandparental heat stress, but only in late-flowering genotypes, and these responses fell within the same range (grandparental: ca. −12 to +2 days; parental: ca. −4 to +2 days) as in our experiment. Our own results confirm that stress exposure can induce earlier flowering also transgenerationally, and thereby contribute to an escape strategy, but that such effects may be restricted to situations with early stress exposure, where plants are still in sensitive developmental stages. Transgenerational variation in flowering time could in principle also be caused by transgenerational variation in germination time (cf. Liu *et al.* 2020). We did not record germination time, but we generally observed very rapid germination after sowing (i.e. cotyledons visible within 1–2 days; personal observation) and are therefore confident that the observed transgenerational effects on flowering time are indeed largely due to variation in developmental rate after germination.

### Genotypic variation in transgenerational plasticity

So far, few studies have investigated intraspecific variation in transgenerational plasticity under stress conditions (Gaudet *et al.* 2011; Suter and Widmer 2013; Nolf *et al.* 2016; Groot *et al.* 2017), and our study provides novel evidence for it. Using nine genetically and morphologically diverse genotypes, we found significant genotype × parental treatment interactions both under control conditions and under renewed stress treatments in the offspring generation. Thus, intraspecific variation in environmentally induced transgenerational responses exists in *A. thaliana*. This genotypic variation among widespread origins suggests evolutionary divergence among populations, which could result from adaptation, genetic drift or both. We used only a single genotype per population, precluding assessment of within-population variation (or constancy) of responses to experimental treatments. However, the genetic diversity within populations of *A. thaliana* is likely very restricted (Bomblies *et al.* 2009), whereas genetic diversity is large among the selected populations (McKhann *et al.* 2004). Therefore, we are confident that the observed patterns reflect evolutionary divergence among populations.

### Relationships with climates of origin and fitness robustness

We found that plasticity in response to heat stress correlated with a range of climate variables from the genotypes’ geographic origins, suggesting that environmental variability at sites of origin might be an important selective factor (Endler 1986) for the evolution not only of within-generation plasticity (Scheepens *et al.* 2018) but also of transgenerational plasticity. Interestingly, these relationships were only found under stressful conditions in the reciprocal experiment but not under stress-free conditions in the common-environment experiment.

One of the observed plasticity–climate relationships was a negative correlation between variation in fecundity and temperature seasonality at sites of origin. Variation in the same trait was also correlated to two other, closely related, climate variables: isothermality, which is the proportion of the diurnal range over the annual temperature range (positive correlation) and annual temperature range (negative correlation). Genotypes from origins with higher temperature seasonality showed a reduced variation in fecundity and thus appear to have evolved a stronger fitness homeostasis in the face of fluctuating temperature conditions, whereas genotypes from origins with more stable temperature regimes evolved to respond more strongly to temperature stress, leading to reduced fitness in our experiments. However, it should be noted that these three correlating climatic variables are year-based, whereas the nine *A. thaliana* genotypes differ in growing-season length and period (see Table 1) and therefore experience only part of the temperature variation captured in these variables, which may not reflect the year-based values. The CV of evapotranspiration was the only growing season-based variable (positively) affecting variation in fecundity, suggesting the opposite that genotypes from more variable environments have reduced fitness stability.

We also observed a positive relationship between plasticity in biomass and precipitation seasonality, i.e. plants from unpredictable precipitation environments responded more strongly to temperature stress. Since biomass and fecundity are strongly positively correlated in *A. thaliana* (Clauss and Aarssen 1994), this plasticity–climate relationship seems to contrast with the above-mentioned negative correlation between variation in fecundity and temperature seasonality. However, precipitation seasonality and temperature seasonality are not correlated in the studied plant origins, so these plasticity–environment correlations may reflect independent evolutionary responses to different aspects of climate variability.

The strongest plasticity–environment correlation was between plasticity in above-ground biomass and latitude: plants from higher latitudes responded less to variation in temperature stress. Since increasing latitude is associated with decreasing precipitation seasonality, the latter may be the underlying driver of this relationship. High precipitation seasonality at low latitudes may have selected for strong biomass responses to temperature stress, possibly because heat and drought are the main drivers terminating growth there and plant size strongly affects evapotranspiration and thus survival. Along the same line, we had expected that flowering time would correlate with latitude or climatic variables, potentially reflecting escape mechanisms under periods of drought (Franks 2011), but no relationships with flowering time were observed.

Population genetic structure could potentially also explain the relationship between plasticity in above-ground biomass and latitude. However, when we included a genetic distance matrix (based on 250 k SNPs; Horton *et al.* 2012) in regressions of plasticity in above-ground biomass with latitude as explanatory factor (using the R package lme4qtl; Ziyatdinov *et al.* 2018), population genetic structure did not explain variation (*P* = 0.55), whereas latitude remained significant (*F* = 44.11, *P* < 0.001).

In the parental experiment (Scheepens *et al.* 2018), we had previously found positive correlations between plasticity and precipitation variability at sites of origin in four out of five traits. We did not find the same relationship in the offspring generation in the current study, even though transgenerational effects were still present in three out of five traits. One possible explanation for this is that the plant responses in the parental generation were passive and/or maladaptive (cf. fitness robustness), and that transgenerational effects caused the offspring generation to respond less in order to retain fitness. We did find correlations between plasticity in plant architecture, above-ground biomass and fecundity and several other climate variables in the reciprocal experiment, indicating a possible adaptive function of these plant responses, and highlighting the general relevance of studying environmental variability for understanding transgenerational plant responses to temperature stress.

We found negative correlations between fitness robustness and plasticity in above-ground biomass, but not in other traits, in both experiments. This is similar to our results from the parental plants (Scheepens *et al.* 2018) and implies that more plastic genotypes show stronger fitness variation in response to (parental and/or offspring) treatments. However, the slopes of these relationships are flatter in offspring compared to parental plants, with fitness robustness values of 0.69–0.95 in the common-environment experiment and 0.72–0.97 in the reciprocal experiment, compared to values of 0.50–0.90 in the parental generation (Scheepens *et al.* 2018). Therefore, the offspring generation, even when under identical stress, shows an overall improved fitness robustness, which may reflect a transgenerational adaptive response to temperature stress.

## Conclusions

Given that changes in temporal environmental variability are an important aspect of climate change, it is important to understand their effects on plants, both in terms of phenotypic plastic responses and of intraspecific evolutionary divergence. To our knowledge, no previous study has tested for transgenerational responses of plants to temporal variability of environmental stresses, rather than their mean changes. We found ample genotypic variation in transgenerational responses to temporal variation in heat stress, suggesting that selection can act on it. Furthermore, plasticity–environment correlations suggest possible adaptations to the environmental variability of plant origins. These findings therefore indicate potential of natural populations as well as of crop varieties to adapt to increasingly variable climates in the future.

## Supporting Information

The following additional information is available in the online version of this article—

Figure S1. The effects of timing and frequency of parental heat stress on the flowering time (days since germination) of *Arabidopsis thaliana* in the common-environment experiment.

Figure S2. Genotypic variation in the effects of parental and offspring heat stress timing on fecundity (number of fruits) in nine *Arabidopsis thaliana* genotypes in the reciprocal experiment.

Table S1. Correlations between climates of origin and phenotypic plasticity across nine *Arabidopsis thaliana* genotypes in the common-environment experiment.

Table S2. Correlations between climates of origin and phenotypic plasticity across nine *Arabidopsis thaliana* genotypes in the reciprocal experiment.

Table S3. Correlations between trait plasticities (CV across all treatments) and fitness robustness (see main text) across nine *Arabidopsis thaliana* genotypes.

Table S4. Mean and SE of the flowering time, plant architecture, above-ground biomass, reproductive allocation and fecundity of *Arabidopsis thaliana* offspring under different parental treatment combinations (parental timing and parental frequency) in the common-environment experiment.

Table S5. Mean and SE of the flowering time, plant architecture, above-ground biomass, reproductive allocation and fecundity of *Arabidopsis thaliana* offspring under different treatment combinations (parental timing and offspring timing) in the reciprocal experiment.

Table S6. Excel file containing the raw data from the common-environment experiment and the reciprocal experiment.

plab064_suppl_Supplementary_Material

plab064_suppl_Supplementary_Table_S6

## Data Availability

The raw data of this publication can be found in the **Supporting Information—Table S6**.

## Notes

**Plants, Ecosystems & Climate. Chief Editor:** Mary Heskel
