# Supplementary material for: Transgenerational effects of temperature fluctuations in Arabidopsis thaliana
Source: AoB Plants. 2021 Oct 11;13(6):plab064. doi: 10.1093/aobpla/plab064 (PMC8691168; doi:10.1093/aobpla/plab064)
Supplement: plab064_suppl_Supplementary_Material [file plab064_suppl_supplementary_material.pdf]

SUPPORTING INFORMATION

Transgenerational effects of temperature fluctuations in *Arabidopsis thaliana*

Figure S1. The effects of timing and frequency of parental heat stress on the flowering time (days since germination) of *Arabidopsis thaliana* in the common-environment experiment. Error bars indicate SE.

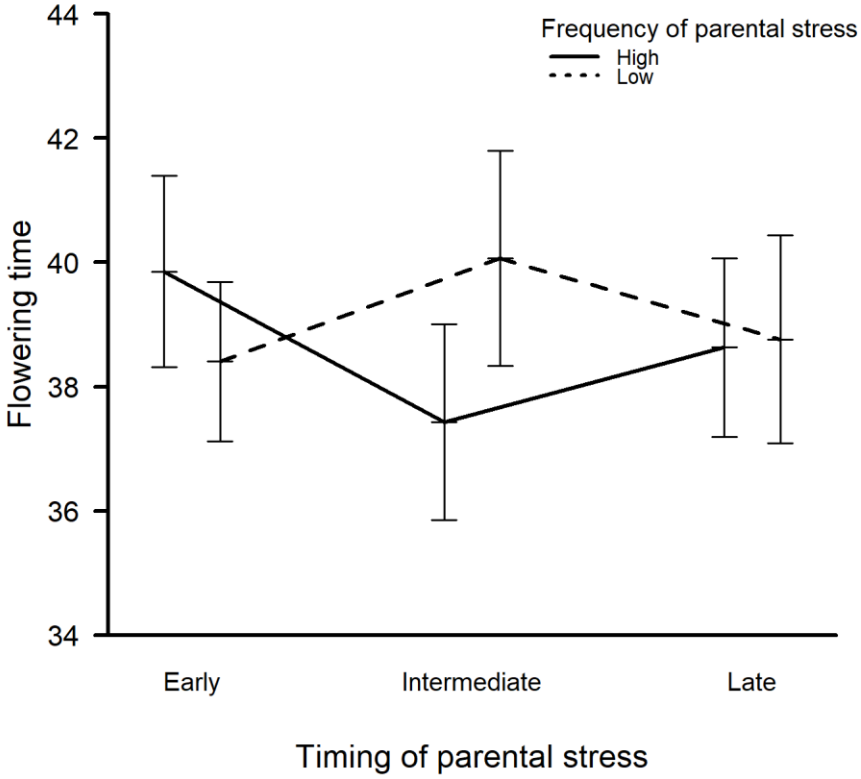

Figure S2. Genotypic variation in the effects of parental and offspring heat stress timing on fecundity (number of fruits) in nine *Arabidopsis thaliana* genotypes in the reciprocal experiment. Error bars indicate SE.

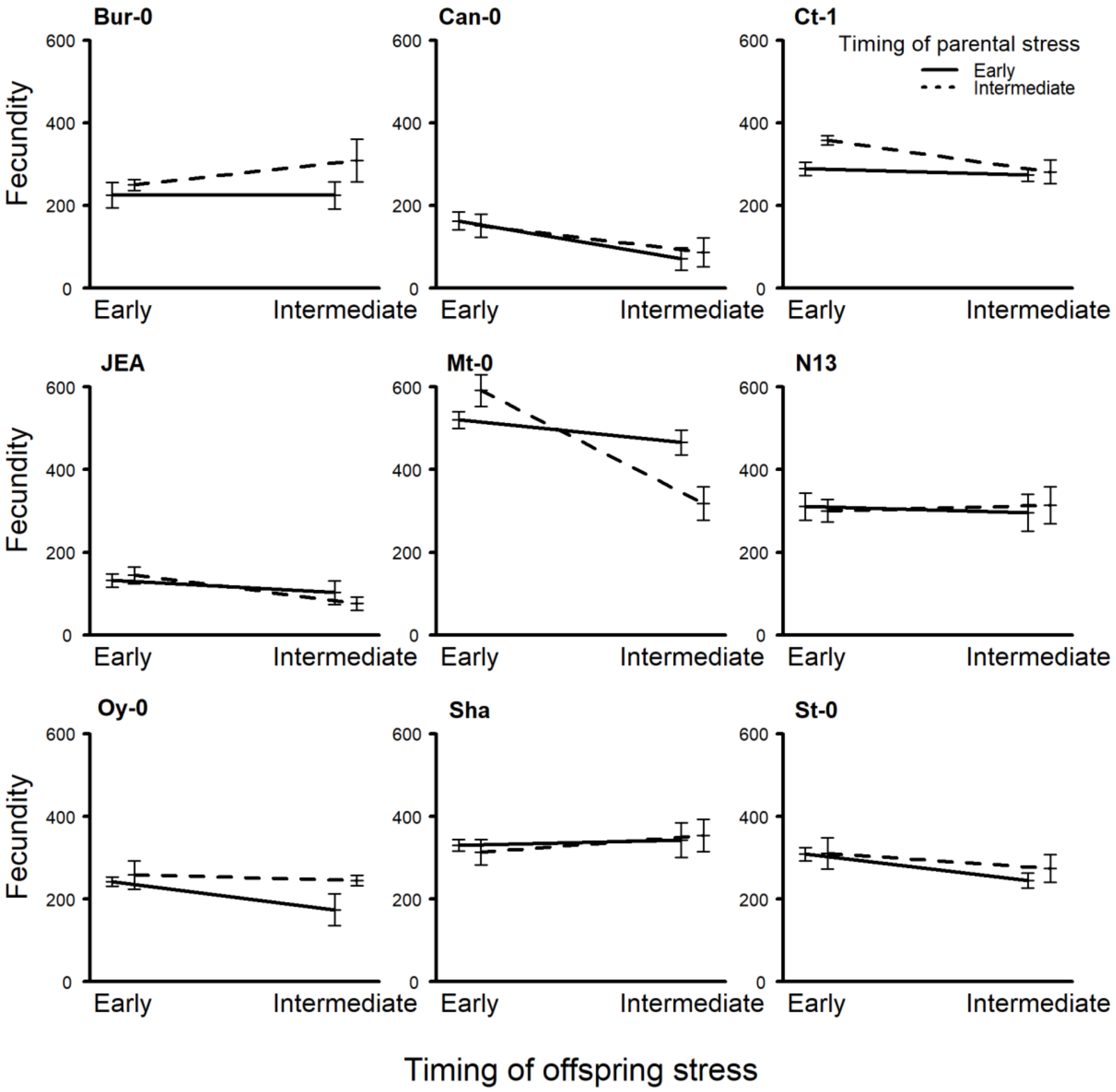

Table S1. Correlations between climates of origin and phenotypic plasticity across nine *Arabidopsis thaliana* genotypes in the common-environment experiment. The *R*-values are Pearson correlation coefficients.

**Climate variables**	**Flowering time**		**Plant architecture**		**Aboveground biomass**	
	R	*P*-value	*R*	*P*-value	*R*	*P*-value
Growing season-based						
Temperature SD	-0.08	0.845	0.48	0.188	-0.12	0.755
Precipitation CV	0.37	0.333	0.00	0.990	-0.51	0.159
Evapotranspiration CV	0.54	0.133	0.15	0.708	0.45	0.222
Climatological Water Deficit CV	-0.19	0.624	-0.31	0.414	-0.02	0.966
Year-based						
Annual Mean Diurnal Temperature Range	-0.14	0.711	0.27	0.481	-0.56	0.113
Isothermality	0.25	0.511	0.37	0.322	-0.50	0.166
Temperature Seasonality (SD)	-0.26	0.492	-0.31	0.412	0.17	0.655
Annual Temperature Range	-0.22	0.567	-0.20	0.615	-0.01	0.983
Precipitation Seasonality (CV)	0.19	0.630	0.21	0.588	-0.39	0.300
Latitude	-0.28	0.470	-0.42	0.266	0.43	0.250

Table S2. Correlations between climates of origin and phenotypic plasticity across nine *Arabidopsis thaliana* genotypes in the reciprocal experiment. The *R*-values are Pearson correlation coefficients. Significant correlations (*P* < 0.05) are in bold.

**Climate variables**	**Flowering time**		**Plant architecture**		**Aboveground biomass**		**Fecundity**	
	R	*P*-value	*R*	*P*-value	*R*	*P*-value	*R*	*P*-value
Growing season-based								
Temperature SD	0.20	0.605	-0.17	0.670	-0.20	0.609	-0.38	0.312
Precipitation CV	0.10	0.795	0.13	0.745	-0.01	0.974	0.13	0.747
Evapotranspiration CV	-0.19	0.629	0.50	0.175	0.47	0.200	0.73	**0.025**
Climatological Water Deficit CV	-0.14	0.713	0.27	0.475	-0.84	**0.005**	-0.58	0.099
Year-based								
Annual Mean Diurnal Temperature Range	0.21	0.589	-0.73	**0.027**	0.32	0.401	-0.24	0.526
Isothermality	0.45	0.225	-0.21	0.593	0.86	**0.003**	0.74	**0.022**
Temperature Seasonality (SD)	-0.37	0.325	-0.32	0.404	-0.63	0.066	-0.87	**0.002**
Annual Temperature Range	-0.28	0.469	-0.49	0.183	-0.45	0.222	-0.82	**0.007**
Precipitation Seasonality (CV)	0.05	0.889	-0.47	0.200	0.85	**0.004**	0.38	0.312
Latitude	-0.30	0.428	0.41	0.278	-0.92	**<0.001**	-0.58	0.104

Table S3. Correlations between trait plasticities (CV across all treatments) and fitness robustness (see main text) across nine *Arabidopsis thaliana* genotypes. The *R*-values are Pearson correlation coefficients.

	**Flowering time**		**Plant architecture**		**Aboveground biomass**	
	R	*P*-value	*R*	*P*-value	*R*	*P*-value
Common-environment experiment	-0.31	0.422	0.11	0.785	-0.79	**0.012**
Reciprocal experiment	-0.47	0.200	-0.15	0.702	-0.71	**0.031**

Table S4. Mean and SE of the flowering time, plant architecture, aboveground biomass, reproductive allocation and fecundity of *Arabidopsis thaliana* offspring under different parental treatment combinations (parental timing and parental frequency) in the common-environment experiment.

	Parental timing	Parental frequency	Mean	SE
Flowering time	Early	Low	38.40	1.28
	Mid	Low	40.06	1.73
	Late	Low	38.76	1.68
	Early	High	39.85	1.54
	Mid	High	37.43	1.57
	Late	High	38.63	1.44
Plant architecture	Early	Low	3.29	0.34
	Mid	Low	2.84	0.34
	Late	Low	2.87	0.30
	Early	High	3.69	0.38
	Mid	High	3.04	0.32
	Late	High	3.08	0.33
Aboveground biomass	Early	Low	684.05	22.91
	Mid	Low	691.39	27.66
	Late	Low	712.02	26.53
	Early	High	710.52	25.28
	Mid	High	690.61	25.21
	Late	High	708.30	27.94
Reproductive allocation	Early	Low	0.63	0.02
	Mid	Low	0.65	0.02
	Late	Low	0.65	0.02
	Early	High	0.64	0.02
	Mid	High	0.65	0.03
	Late	High	0.63	0.03
Fecundity	Early	Low	213.30	13.67
	Mid	Low	213.57	14.74
	Late	Low	229.41	12.41
	Early	High	219.44	14.07
	Mid	High	224.73	15.94
	Late	High	230.13	16.14

Table S5. Mean and SE of the flowering time, plant architecture, aboveground biomass, reproductive allocation and fecundity of *Arabidopsis thaliana* offspring under different treatment combinations (parental timing and offspring timing) in the reciprocal experiment.

	Parental timing	Offspring timing	Mean	SE
Flowering time	Early	Early	35.21	1.38
	Mid	Early	38.03	1.63
	Early	Mid	35.90	1.20
	Mid	Mid	37.71	1.76
Plant architecture	Early	Early	2.28	0.27
	Mid	Early	2.34	0.26
	Early	Mid	3.07	0.32
	Mid	Mid	2.96	0.31
Aboveground biomass	Early	Early	756.53	15.93
	Mid	Early	763.55	19.27
	Early	Mid	655.53	25.23
	Mid	Mid	663.57	24.37
Reproductive allocation	Early	Early	0.71	0.02
	Mid	Early	0.71	0.02
	Early	Mid	0.60	0.02
	Mid	Mid	0.61	0.02
Fecundity	Early	Early	280.01	14.27
	Mid	Early	298.83	17.68
	Early	Mid	248.12	17.17
	Mid	Mid	250.17	15.83
